# Fitness Cost of *Litomosoides sigmodontis* Filarial Infection in Mite Vectors; Implications of Infected Haematophagous Arthropod Excretory Products in Host-Vector Interactions

**DOI:** 10.1155/2013/584105

**Published:** 2013-09-09

**Authors:** Adélaïde Nieguitsila, Roger Frutos, Catherine Moulia, Nathaly Lhermitte-Vallarino, Odile Bain, Laurent Gavotte, Coralie Martin

**Affiliations:** ^1^UMR 7245 MCAM MNHN CNRS, Muséum National d'Histoire Naturelle, 61 rue Buffon, CP52, 75231 Paris Cedex 05, France; ^2^UMR 5554 ISEM CNRS, Université Montpellier 2, Place Eugène Bataillon, 34095 Montpellier, France; ^3^UMR 5236 Centre d'Études d'Agents Pathogènes et Biotechnologies pour la Santé (CPBS), 34095 Montpellier, France

## Abstract

Filariae are a leading cause of infections which are responsible for serious dermatological, ocular, and vascular lesions. Infective third stage larvae (L3) are transmitted through the bite of a haematophagous vector. *Litomosoides sigmodontis* is a well-established model of filariasis in the mouse, with the vector being the mite *Ornithonyssus bacoti*. The aim of the study was to analyse the filarial infection in mites to determine the consequences of filarial infection in the blood-feeding and the reproduction of mites as well as in the regulation of vector-induced inflammation in the mouse skin. Firstly, L3 are unevenly distributed throughout the host population and the majority of the population harbours a moderate infection (1 to 6 L3). Filarial infection does not significantly affect the probing delay for blood feeding. The number of released protonymphs is lower in infected mites but is not correlated with the L3 burden. Finally, induced excreted proteins from infected mites but not from uninfected mites stimulate TNF-**α** and the neutrophil-chemoattractant KC production by antigen-presenting cells (APCs). Altogether, these results describe the modification of the mite behavior under filarial infection and suggest that the immunomodulatory capacity of the mite may be modified by the presence of the parasite, hindering its defensive ability towards the vertebrate host.

## 1. Introduction

Arthropod-borne diseases are a major concern worldwide. Among them, filarial nematodes are transmitted by haematophagous vectors and affect humans and domestic and wild animals [1]. Numerous filarial species are transmitted by different vectors (hard and soft ticks, black flies, *Chrysops*, or mosquitoes). Their L3 are injected into the host during a blood meal of the arthropod vector and develop into adult worms which release microfilariae into either the skin (e.g., *Onchocerca* spp.) or the blood (e.g., *Brugia* spp. and *Dirofilaria* spp.). As for other vectored parasites, the invertebrate blood meal is a key time in their life cycle.

*Litomosoides sigmodontis* (Chandler, 1931) is a well-known murine model of filariasis with adult worms residing in the pleural cavity of its rodent host [2] and blood microfilariae [3]. This filarial species is experimentally maintained through the passage in its natural host, the cotton rat *Sigmodon hispidus* [4], or in the jird *Meriones unguiculatus* [5]. *L. sigmodontis* is transmitted by *Ornithonyssus bacoti* (first described by Hirst, 1913; Macronyssidae family), a solenophagous mite with needle-like mouth parts.

The life cycle of *O. bacoti* includes five stages [6]. Initially, eggs hatch in hexapod larvae which will moult into protonymphs about 24 hours later and will need a blood meal before moulting into deutonymphs. Deutonymphs do not need a blood meal; they moult into male or female adults within 24 to 36 hours. Fertilisation occurs within 24 to 48 hours after the adult mites have emerged and female adults need to take a blood meal in order to lay their eggs. The whole cycle takes 11 to 14 days. Nevertheless, the feeding behaviour of *O. bacoti* is poorly documented. It is known that the adult mites feed during a relatively brief period (up to 20 minutes) before they leave the host [7]. However, mites frequently interrupt their feeding [8]. Thus the minimal time required to obtain maximum feeding suggests hours of contact between mites and the vertebrate [9]. Blood is then digested in the haemocoel cavity of the mites.

 The passage of *L. sigmodontis* microfilariae from vertebrate host into *O. bacoti* is influenced by many factors such as the local density of the microfilariae in the host; that is, the number of ingested microfilariae is lower in case of high microfilaraemia, a regulation process named limitation [5]. The microfilariae can develop in various tissues of the mite such as the interstitial tissue, the salivary glands, and the coxal glands. There, they moult into stage 2 larvae L2 (within 5–7 days), and then into L3 within 9–11 days, and infective larvae migrate back to the haemocoel [10, 11] from where they will escape and reach the vertebrate host skin during a blood meal of the mites. 

The skin constitutes a key interface in the development of vector-borne diseases, and vectors' saliva is essential for interactions between arthropod, parasite, and host. Even though the precise mechanisms are still poorly understood, it has been established that, during a blood meal, vector saliva can activate a complex cascade of interacting events that eventually results in local and systemic inflammatory and immune reactions in the host. Saliva can affect both the innate and adaptive immunity of the vertebrate host skin [12, 13] and some key mediators of innate immunity such as antimicrobial peptides (e.g., cathelicidin and defensin families) have been identified as important initiators of skin inflammation [14, 15]. Pathogens transmitted by these vectors interact with both saliva components and host mediators, taking advantage of the modified host physiology in order to become established [16–18]. 

 Although *L. sigmodontis* is a well-established model of filariasis in vertebrates, its relationships with the invertebrate vector are mainly unknown. The present study was undertaken to compare the blood meal behaviour of the uninfected and infected mites, their reproduction, and the host APC's inflammatory response.

## 2. Material and Methods

### 2.1. Ethics Statement

All experimental procedures were carried out in strict accordance with EU Directive 2010/63/UE and the relevant National Legislation (French Décret no. 2013-118 from February 1, 2013). Protocols were approved by the ethical committee “Cuvier” of the “Museum National d'Histoire Naturelle” (no. 68-002).

### 2.2. Parasite Maintenance

Maintenance of the filaria *L. sigmodontis* (Chandler, 1931) and the recovery of infective larvae (L3) from the mite vector, *O. bacoti*, were carried out as previously described [3, 5]. Briefly, 300 one-week-starved mites were allowed to blood-feed on infected* M. unguiculatus* jirds (700–2000 microfilariae/mm^3^ in the peripheral blood) overnight. Then blood-fed mites were recovered and kept at 28°C and the 80% relative humidity to allow the *L. sigmodontis* development from microfilariae into L3. Jirds were obtained from Charles River France and maintained in the MNHN animal facilities. 

Six-to-eight-week-old ICR outbred female mice were used for infection protocols. Six-to-eight-week-old female BALB/c mice were used for APC generation and immune analysis. Both strains of mice were obtained from Harlan France and maintained in the MNHN animal facilities.

### 2.3. Infection Protocols

1. Five groups of 12-day infected mites (*n* = 22–50) fed on different jirds (700–2000 microfilariae/mm^3^ in the peripheral blood) were dissected under a binocular microscope. Infected larvae (L3) were recovered and counted.
2. Groups of ten 12-day infected mites or groups of ten uninfected mites were allowed to take blood on the left lumbar area of an ICR mouse. Mites were left in contact with the mouse for either 1.5 h, 6 h, or 12 h. The experiment was repeated six times, each on different mice. Blood meal was confined to a circle of 0.5 cm diameter. Indeed, 10 mites were gathered in the lid of a 2 mL Eppendorf tube, which then was fixed with plaster to the shaved skin surface of anesthetised ICR mouse. The number of blood-fed mites was evaluated in each lid. Then each infective blood-fed and blood-unfed mite was dissected and the number of infective larvae (L3) was counted.

### 2.4. Production and Quantification of Protonymphs

Mites were either fed on infected or uninfected jirds overnight. Mites were then randomly selected on each jird and placed individually in glass tubes at 28°C and 80% relative humidity. Twelve days later, when microfilariae had become L3, the number of protonymphs was evaluated by observation under a binocular microscope. Mites blood-fed on infected jird were necropsied and the number of L3 was determined. 

### 2.5. Induction and Quantification of Mite-Excreted Proteins

A method of collecting saliva from *Anopheles* spp. was adapted to mites to stimulate the excretion process [19]. Groups of 100 12-day-infected or -uninfected mites were placed for 2 hours in 24-well plates containing 200 *μ*L of a saline solution (Hepes 10 mM, NaCl 150 mM, and EDTA 5 mM; pH 7.2) at two different temperatures (25°C and 37°C) to stimulate salivary and extrasalivary excretions. Supernatants were collected, and quantification of total proteins was determined using a colorimetric detection based on bicinchoninic acid (Pierce BCA protein assay) [20].

### 2.6. Generation of APC from Murine Bone Marrow

APCs were generated *in vitro* from bone marrow (BM) cells from 6-week-old female BALB/c mice (*n* = 10). Briefly, both femurs from each mouse were flushed with RPMI-1640 to release the BM cells which were then cultured in 10 cm tissue culture dishes in 10 mL RPMI-1640 supplemented with 10% heat-inactivated foetal calf serum (FCS), 100 *μ*g/mL of penicillin, 100 *μ*g/mL of streptomycin, 1% L-glutamine, 1% nonessential amino acids (NEAA), 1% sodium pyruvate (Eurobio, France), and 25 ng/mL murine Granulocyte-Macrophage Colony-Stimulating Factor (GM-CSF, Peprotech). On day 3, half of the supernatants were gently removed and replaced with the same volume of culture medium. On day 6 adherent and nonadherent cells detached with 5 mL 2 mM EDTA were collected; cells were centrifuged at 400 ×g at 4°C, supernatants were discarded and replaced with new supplemented medium, and cell suspensions were plated out in two 10 cm tissue culture dishes. On day 9 adherent cells were detached, and counted and then evaluated by flow cytometry (30% of cells were CD11c positive and 65% were F4/80 positive).

### 2.7. APC Activation and Determination of Cytokine Concentrations by ELISA

APCs (10^6^ cells/mL) were seeded in 96-well plates in duplicate and stimulated for 24 h at 37°C and 5% CO_2_ with 10 *μ*g/mL of supernatants containing saline vehicle or induced-excreted proteins from infected or uninfected mites. Concentrations of tumour necrosis factor-alpha (TNF-*α*) and chemokine CXCL1 (KC) were measured in the APC supernatants by enzyme-linked immunosorbent assay (ELISA) using ELISA development kits purchased from eBioscience and PeproTech France, respectively. These assays were performed according to the manufacturers' recommendations, and results are expressed as pg/mL. ABTS peroxidase substrate (KPL, Gaithersburg, USA) for PeproTech kit and tetramethylbenzidine (TMB) stopped with a 2 N H_2_SO_4_ solution for eBioscience kit were used as substrates, respectively. ABTS peroxidase substrate contains 2,2′-azino-di(3-ethylbenzthiazoline-6-sulfonate) at a concentration of 0.3 g/L in a glycine/citric acid buffer. The absorbance was read at 405 nm or 450 nm wavelength, respectively, with a Labsystems Multiscan MS ELISA reader. The detection limit was 8 pg/mL for both KC and TNF-*α*.

### 2.8. Statistics

Statistical analyses were performed using the software StatView 5.0.1 (SAS Institute 1998) and the PRISM 5 programme (GraphPad Software, Inc., La Jolla, USA). Data from separate experiments were pooled when homoscedasticity was significantly tested with the Bartlett test. The assumption of normality was tested with Kolmogorov-Smirnov test. If the interaction term was significant, comparisons using *t*-tests or ANOVA tests coupled with post hoc Bonferroni adjustments were realized. Otherwise, nonparametric tests were used. 

The differences between infection status of mites and those between times as well as the differences between feeding status of mites and those between times were analyzed by a two-way analysis of variance. Further comparisons were assessed by Bonferroni's multiple comparison test. The number of protonymphs was tested using Mann-Whitney *U* test. The correlation between the number of protonymphs and the number of L3 was determined using the Spearman correlation test. Protein excretions were analysed using *t*-test and ELISA results were studied using one-way ANOVA followed by Bonferroni's multiple comparisons posttest.

## 3. Results

### 3.1. Population Aspects of Filarial Infection in the Mite Vector

**Figure 1:**
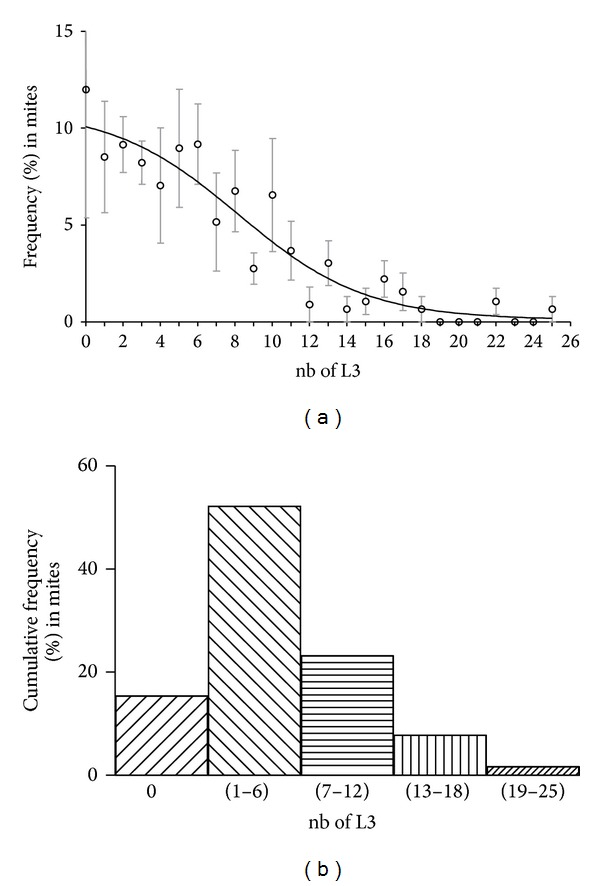
Distribution of *L. sigmodontis* infective larvae in *O. bacoti *mites. Five groups of 12-day infected mites (*n* = 22–50) fed on different jirds were dissected. Infected larvae (L3) were recovered and counted. (a) The frequency of L3 in mites was evaluated and the distribution analysed; a curve fit using nonlinear fitting is shown; each point and error bars are mean ± SEM. (b) Five classes of L3 are made and the cumulative frequency of L3 in mites is presented.

To determine the average level of *L. sigmodontis* L3 infection in the *O. bacoti* mites as well as the parasite frequency distribution in a mite population, 5 groups of infected mites were analysed for their content of L3. First, it appears that L3 are unevenly distributed throughout the host population (Figure 1(a)). A proportion of mites do not have L3 even if they blood-fed on microfilariae-infected blood of jirds (Figure 1(b)). Most of the mites (52%) carried between 1 and 6 L3 (Figure 1(b)). This proportion then decreased consistently to almost zero in mites carrying more than 19 L3. The average number of L3 per mite is 5.3 ± 0.36.

### 3.2. Time to Blood-Feed Is Similar in Mites Irrespective of Their Infectious Status

**Figure 2:**
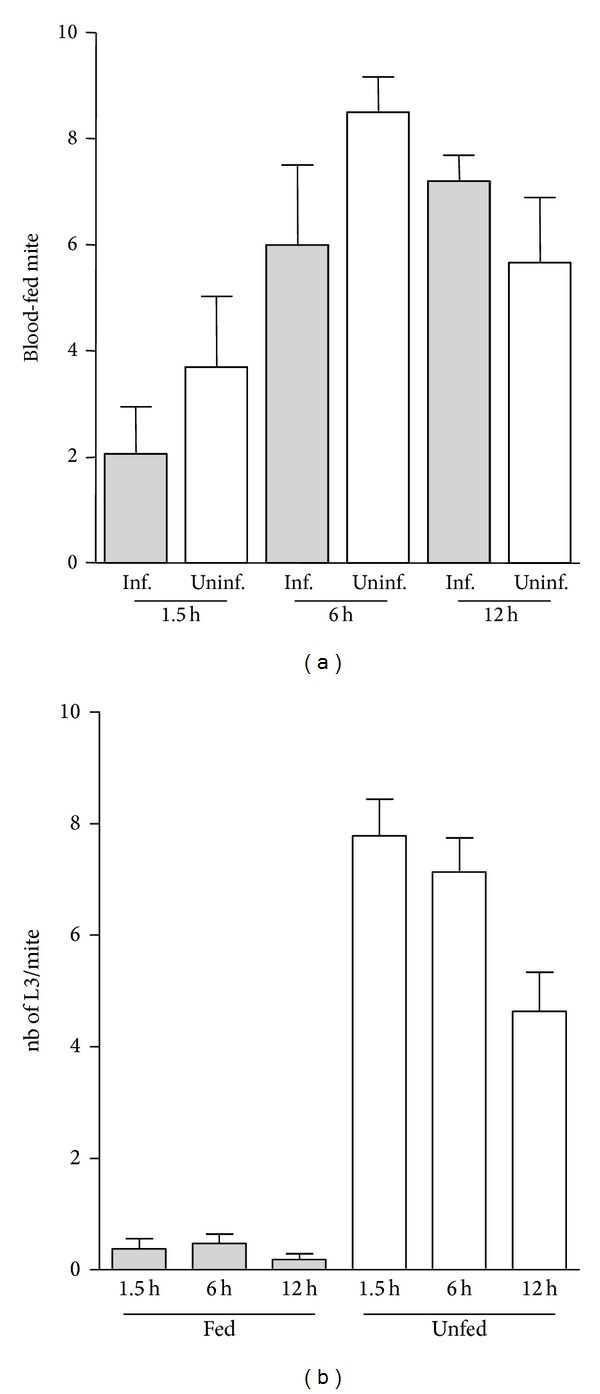
Kinetics of blood-feeding and rate of *L. sigmodontis* infection in mites. One-week starved adult mites (*n* = 300) were allowed to feed on either an infected jird or an uninfected jird; 12 days later, that is, when larvae are L3 in mites, smaller groups of these mites were allowed to take blood on an ICR uninfected mouse for various lengths of time (1.5 h, 6 h, or 12 h). (a) Kinetics of blood-feeding in mites: for each time point, 10 uninfected (Uninf.) or 10 infected (Inf.) mites were blood-fed on ICR mice. The number of effective blood-fed mites (fed) was evaluated in each group. The experiment was repeated 6 times and pooled. Infection status and time effects were not significant (two-way analysis of variance). Results are expressed as mean ± SEM. (b) Rate of L3 infection in mites: for each time point, 10 mites from the group fed on infected jirds were blood-fed on ICR mice, and then fed and unfed mites were separated. Each individual mite was necropsied and the L3 were searched for and isolated. The experiment was repeated 6 times and the data were pooled. Feeding status was significant (two-way analysis of variance). A time effect was significant neither in “unfed” nor in “fed” group (Bonferroni's multiple comparison test). Results are expressed as mean ± SEM.

*O. bacoti* mites are known to blood-feed for a relatively brief period (a few minutes, up to 20 min) which can be frequently interrupted [7]. Thus the contact time between the mites and the vertebrate can be many hours. To evaluate the consequences of filarial infection in this blood-feeding behaviour, three time points (1.5 h, 6 h, and 12 h) have been analysed with respect to the number of blood-fed mites and to the optimal time of presence on the host for blood-feeding. It appears that the best length of time for uninfected or infected mites to take blood is 6 hours; however, not all of the mites are able to take a blood meal; this is demonstrated by the observation that a maximum of 85% of mites were blood-fed (Figure 2(a)). Although the proportion of blood-fed infected mite is lower at 1.5 h and 6 h, the infectious status of mites does not significantly modify this proportion at any time point. 

After blood-feeding, almost all L3 were found to be transmitted to the mouse (Figure 2(b)) whatever the time length of contact was between the mite and its host.

### 3.3. The Number of Protonymphs Is Lower in Infected Mites but Is Not Correlated with the L3 Burden

**Figure 3:**
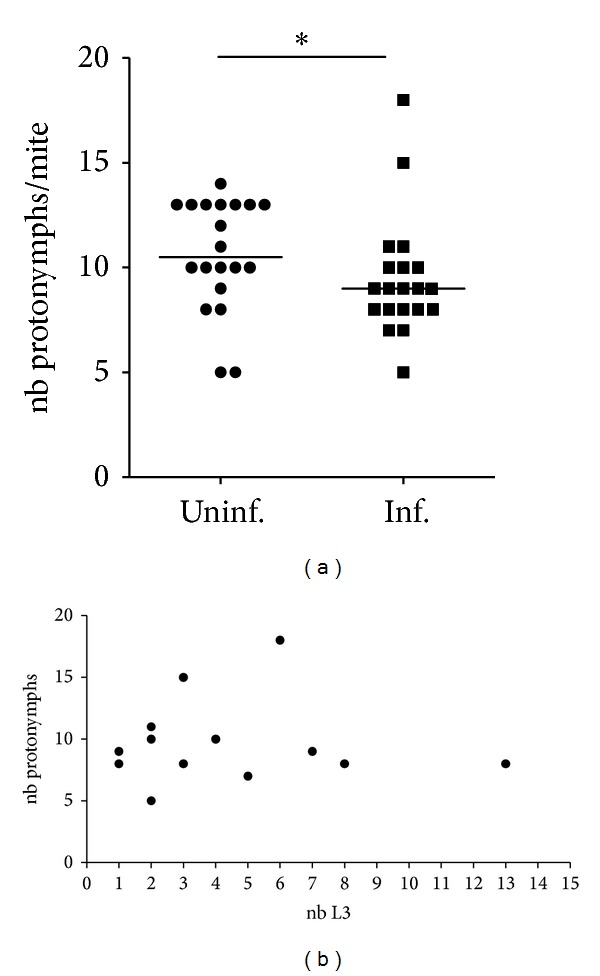
Effect of the filarial infection on the production of protonymphs in mites. Two groups of 1-week starved mites (*n* = 300) were allowed to feed on infected or uninfected jirds for 12 hours; 20 infected females (Inf. mite) and 20 uninfected females (Uninf. mite) were randomly isolated and stored individually in glass tube for 12 days. The number (nb) of protonymphs per tube, thus per mite, was counted (a) as well as the number of L3 per infected mite. The production of protonymphs was different between infected and uninfected mites (Mann-Whitney *U* test, *P* = 0.0387). Median is shown. (b) The number of protonymphs and the number of L3 illustrated are not correlated. Correlation was determined using the Spearman correlation test (*r* = −0.093).

To determine whether the filarial infection has an impact on the fitness of the mites, the individual fecundity was evaluated by counting the number of protonymphs produced. Firstly, there was a significant reduction in the number of protonymphs produced by mites when they were infected by *L. sigmodontis* (Figure 3(a)). Secondly, there was no correlation between the number of protonymphs and the number of L3 per mite (Figure 3(b)).

### 3.4. Only Excretory Products from Infected Mites Stimulate KC and TNF-*α* Production by APC

**Figure 4:**
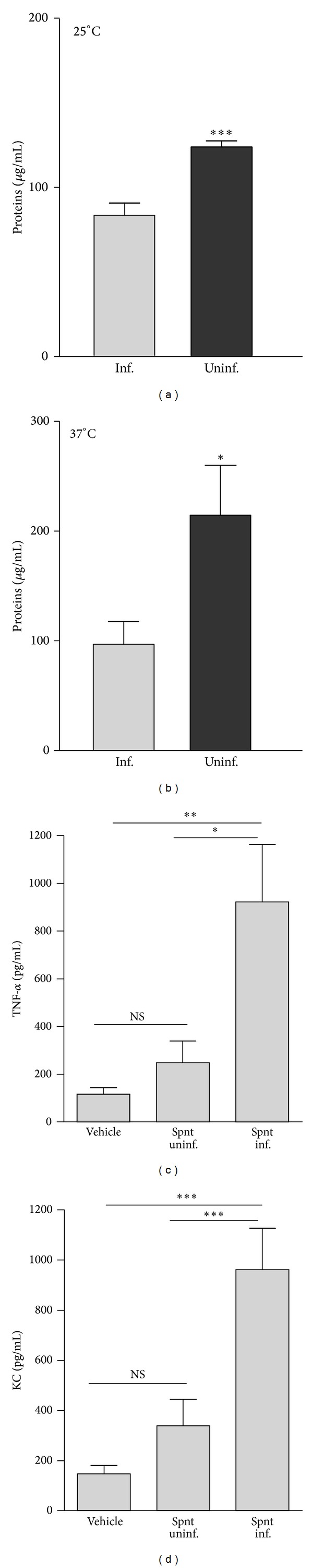
Excretory compounds from infected mites only stimulate KC and TNF-*α* production by APC. ((a) and (b)) 12-day infected or uninfected mites were washed twice in PBS 1X and groups of 100 mites were placed for 2 hours at 25°C or 37°C in 24-well plates with 200 *μ*L of saline solution to stimulate salivary excretion. Supernatants were collected and the quantification of proteins was evaluated by the Bradford method. Uninfected individuals produced more proteins than uninfected at 25°C (Student's *t*-test, *P* = 0.0009) as well as at 37°C (Student *t*-test, *P* = 0.0245); results are expressed as mean ± SEM. ((c) and (d)) Bone-marrow-derived APCs were seeded into 96-well plates (10^6^/well) in 200 *μ*L buffer (RPMI 1640, 10% FCS, 100 U P/S, 1% L-glutamine, 1% NEAA, 1% sodium pyruvate) and cultured for 24 hours (5% CO_2_; 37°C) with saline solution (vehicle), and supernatant from infected mites (Spnt Inf.), or supernatant from uninfected (Spnt Uninf.) mites; both excretory solutions were diluted in saline solution to 10 *μ*g/mL total proteins. Levels of TNF-*α* and KC were measured by ELISA in the culture supernatant. A significant increase of TNF-*α* or KC (one-way ANOVA followed by Bonferroni post hoc test; *P* < 0.0001 and *P* = 0.0012, resp.) was observed in culture supernatants after addition of infected mites excretory solution; results are expressed as mean ± SEM.

To evaluate the general responsiveness of mites to a filarial infection, the release of their excretory products was stimulated by adapting a method of collecting saliva from *Anopheles* [19]. The concentration of total proteins in supernatants containing induced-excreted proteins from infected mites was lower than that from uninfected mites (Figures 4(a) and 4(b)), irrespective of the 2 hour culture temperature, that is, 25°C (Figure 4(a)) or 37°C (Figure 4(b)). In addition, for the same amount of proteins, these excretory products differentially stimulated TNF-*α* and the neutrophil-chemoattractant KC production by APCs. Indeed, excretory products from infected mites induced APCs to release TNF-*α* (Figure 4(c)) and KC (Figure 4(d)) but not those from uninfected mites.

## 4. Discussion

 The life cycles of parasites involve complex interactions between the parasites and their hosts. For vectored parasites, the complexity is increased by the interactions between the vector (usually invertebrate) and the vertebrate host. Regarding filariasis, the vector plays an important role in filarial transmission control [21] throughout its transmission dynamics, at two levels, the uptake of microfilariae [22–25] and the transmission of infective larvae. Vector fitness is thus a crucial factor of vectorial capacity and filarial transmission. However, the question of whether filariae affect the fitness of vectors they infect is largely unknown. Here we have shown in the most studied model of filariasis, that is, *Litomosoides sigmodontis*, that the filarial infection has an effect on different vector traits such as vectorial reproduction and the vertebrate host immune reaction during blood-feeding of infected vectors.

 This study also investigated the aggregation of parasites in the vector and the blood-feeding behaviour of the vector. Parasite distribution in host populations follows ecological rules leading to a distribution best fitting to negative binomial. Indeed the aggregation of parasites in the host population is influenced by various parameters such as host physiology, immunology, behavior, and spatiotemporal position implying a variable susceptibility to infection. As expected, the distribution of *L. sigmodontis* in its vector shows different infection patterns (Figure 1); a tiny proportion of individuals is heavily infected (more than 20 L3 per vector), and another small proportion is devoid of larvae and the majority of the population harbours a moderate infection (between 1 and 6 L3 per vector). These differences could be the result of variations in blood volume taken by the mite, the location on the host where the blood was taken, or the immune response of the vector [26] such as a modification in the haemocyte population [27] which would block the filarial migration or maturation. No evidence of a detrimental effect on the vectors' survival linked to the parasitic burden was observed. Such quantification is essential in order to determine the dynamics of filarial infection in vector populations and then to model its epidemiology. However, our results reveal that *L. sigmodontis *infection is costly for *O. bacoti* offspring by significantly reducing the number of individuals emerging as protonymphs (Figure 3(a)). Unexpectedly, the parasitic load (Figure 3(b)) does not significantly influence the offspring size and only the presence of parasite (from only one L3) has an effect. This implies an absence of a direct metabolic impact and suggests that this fitness reduction is directly linked to the response of *O. bacoti* to the infection, such as a physiological modification or immune response detrimental to egg development, as was observed in other infected vectors [28]. 

 The blood-feeding event is the unique time in the filarial life cycle which allows the transmission of the parasite between the vector and the host. The efficiency of the passage is influenced by many factors such as the distribution and periodicity of microfilariae in capillaries for vector infection, blood-feeding duration, and frequency of transmission from vector to vertebrate. This last step is highly dependent on the contrasting biological features of the vectors, discriminating free-living haematophagous insects (such as mosquitoes, sandflies, and mites) from Argasid (soft) and Ixodid (hard) ticks. Indeed it is possible that repeated or single blood meals as well as intervals between them have a direct impact on their role as vectors [29]. The *O. bacoti* behaviour implies a relatively long time (hours) before probing and one or a few short (minutes) blood meals [7]. We demonstrated (Figure 2(a)) that a filarial infection does not significantly affect the probing delay of *O. bacoti*. However, it seems that with a short contact time between infected mites and their host, the success of blood-feeding is slightly lower than that for uninfected mites. In other vector-host couples, results can vary, such as in *Plasmodium falciparum*-infected *Anopheles gambiae* s.I. in which mosquitoes probe more often and for a longer period of time than their uninfected counterparts [30]. In addition, it appears (Figure 2(b)) that infected mites release nearly all L3 during their subsequent blood meal whatever the contact time was between the vector and its host. The transfer of almost all L3 from the vector to the host has also been demonstrated in the transmission of the human filariae *Wuchereria bancrofti* by the vector *Aedes polynesiensis* [31]. However, the nature of the vector greatly influences this transfer as only a small percentage of *W. bancrofti* L3 was transferred by the vector *Anopheles gambiae* [32]. A selective release of pathogens has been demonstrated for apicomplexa such as *Plasmodium* spp., bacteria, and viruses: *Plasmodium*-infected mosquitoes may need more probing attempts before being successful and thus may enhance transmission as some sporozoites are released at each probe [33]. However the 100% success of L3 in exiting the mite could be explained by their strong mobility due to a powerful musculature. In addition, considering that there are already two bottlenecks in any filarial cycle, that is, passage of microfilariae through the mouthparts of the mite [25, 34] and the high destruction of L3 once inside the skin of the vertebrate [35, 36], adaptive mechanisms may have facilitated this part of the cycle.

 Vector saliva contains a large range of compounds that modify the host skin's microenvironment [37]. The combination of vasodilators, antihaemostatic, immunosuppressive, and anti-inflammatory molecules released by vectors [38–41] is potentially beneficial to both the vector and the pathogen. During *O. bacoti* blood-feeding, the short duration of probing associated with such a cocktail of compounds could drastically reduce the potentiality of host immune response and thus could enhance the capacity of the vector to take blood with less frequent interruptions due to inflammation-induced itching. However, parasites are known to monitor their environment in both their host and vector, allowing them to adapt their developmental cycles and to counteract any unfavourable conditions they encounter. For example, blood vascular permeability induced by local inflammation can be advantageous for parasites in facilitating their entry into the host. During the early phase of the *L. sigmodontis* infection, the inflammation in the skin favours the lymphatic drainage of the L3 and their subsequent establishment in the pleural cavity of their murine host. The thymus and activation-regulated chemokine (TARC/CCL17), which is produced by dendritic cells (DCs) upon microbial challenge is involved in these early immune responses limiting filarial parasite invasion into the host [42]. A genetic deficiency of CCL17 or the administration of anti-CCL17 antibodies results in mast cell accumulation and degranulation, enhanced vascular permeability, drainage of interstitial fluid and an increased filarial infection. Higher quantities of pro-inflammatory cytokines TNF-*α* and KC were released by APC stimulated by excretory products from L3-infected mites. TNF-*α* mediates neutrophil activation, and chemokines such as KC are rapidly released under inflammatory situations and play a major role in inducing neutrophil influx [43]. Neutrophils are known to play a prominent role in the initial phase of infection by vector-borne pathogens such as filariae and *Leishmania* [44]. 

 In conclusion, the relationship between *L. sigmodontis* and its vector *O. bacoti* and its murine host could be of particular interest for the study of interactions between vectors and vector-borne parasites as well as for the interface with their vertebrate host.
